# Uncoupling Thermotolerance and Growth Performance in Chinook Salmon: Blood Biochemistry and Immune Capacity

**DOI:** 10.3390/metabo11080547

**Published:** 2021-08-19

**Authors:** Ronald Lulijwa, Tim Young, Jane E. Symonds, Seumas P. Walker, Natalí J. Delorme, Andrea C. Alfaro

**Affiliations:** 1 Aquaculture Biotechnology Research Group, School of Science, Faculty of Health and Environmental Sciences, Auckland University of Technology, Private Bag 92006, Auckland 1142, New Zealand; rlulijwa@gmail.com (R.L.); andrea.alfaro@aut.ac.nz (A.C.A.); 2 National Agricultural Research Organisation (NARO), Rwebitaba Zonal Agricultural Research and Development Institute (Rwebitaba-ZARDI), Fort Portal P.O. Box 96, Uganda; 3 The Centre for Biomedical and Chemical Sciences, School of Science, Auckland University of Technology, Auckland 1142, New Zealand; 4 Cawthron Institute, Nelson 7010, New Zealand; seumas.walker@cawthron.org.nz (S.P.W.); natali.delorme@cawthron.org.nz (N.J.D.)

**Keywords:** king salmon, *Onchorynchus tshawytscha*, thermal stress, summer mortality, marine heatwaves, aquaculture, fish health, immunology, biomarkers, biochemical profiles

## Abstract

Ocean warming and extreme sea surface temperature anomalies are threatening wild and domesticated fish stocks in various regions. Understanding mechanisms for thermotolerance and processes associated with divergent growth performance is key to the future success of aquaculture and fisheries management. Herein, we exposed Chinook salmon (*Oncorhynchus tshawytscha*) to environmentally relevant water temperatures (19–20 °C) approaching their upper physiological limit for three months and sought to identify blood biomarkers associated with thermal stress and resilience. In parallel, blood biochemical associations with growth performance were also investigated. Temperature stress-activated leukocyte apoptosis induced a minor immune response, and influenced blood ion profiles indicative of osmoregulatory perturbation, regardless of how well fish grew. Conversely, fish displaying poor growth performance irrespective of temperature exhibited numerous biomarker shifts including haematology indices, cellular-based enzyme activities, and blood clinical chemistries associated with malnutrition and disturbances in energy metabolism, endocrine functioning, immunocompetence, redox status, and osmoregulation. Findings provide insight into mechanisms of stress tolerance and compromised growth potential. Biochemical phenotypes associated with growth performance and health can potentially be used to improve selective breeding strategies.

## 1. Introduction

Chinook salmon (*Oncorhynchus* *tshawytscha*) are large anadromous fish native to North America and regions in the northwestern Pacific. Valued for their high-quality fillet, global fishery landings peaked in the late 1980s, but have since declined markedly to less than 5000 tonnes in 2019 [1]. Chinook salmon were introduced to New Zealand more than a century ago, and the country’s development of a successful aquaculture industry now provides around 80% of the world’s supply [1]. Salmon are reared only in the South Island of New Zealand where cooler water temperatures are more favourable. However, chronic and acute water temperature variations associated with climate change are starting to pose a significant threat to this industry.

Sea surface temperatures (SSTs) around New Zealand have been rising at a rate of approximately 0.2 °C per decade over the past forty years, and end-of-century forecasts predict a 1.1–2.5 °C increase above the current average [2,3]. Exacerbating the effects of this relatively rapid thermal rise on ecosystems are the increasing incidences of marine heatwaves during the summer months [4,5]. As examples, New Zealand salmon farmers reported substantial stock losses (ca. 20%) over the 2017/2018 and 2018/2019 austral summers due to unprecedented SSTs reaching 2–5 °C above the species’ preferred upper threshold (17 °C) where peak health and growth can be maintained [6,7,8,9]. Temperature-stressed salmon generally have increased metabolic rates and somatic maintenance costs, grow slower with lower condition factors, have suppressed appetite, experience reproductive issues, display compromised immunity, and exhibit increased sensitivity to other stressors [10,11,12,13,14]. However, plasticity in thermotolerance and growth performance is well documented among fishes [13], which affords an optimistic outlook to build potential thermotolerance into New Zealand’s Chinook salmon stocks.

Some individual fish seem better equipped at dealing with stresses than others, and the appearance of divergent growth phenotypes within cohorts is relatively common [15,16,17,18]. Confronted with rapid global change, understanding what makes wild and domesticated fish, at individual and population levels, more or less likely to perform well under dynamically shifting conditions has major ecological and economic implications. Unravelling biological mechanisms underpinning thermotolerance in fish is crucial to developing mitigation strategies to safeguard and futureproof the salmon aquaculture sector.

Accordingly, we performed an elevated temperature challenge on commercial stocks from two of New Zealand’s selective breeding programs to establish thermotolerance trait heritability based on genetic associations with survival and growth performance (unpublished). The current study extends this research by investigating the influence of thermal stress on a targeted suite of 47 blood parameters with potential clinical significance, and their associations with highly divergent growth trajectories. We achieved this by selectively sampling individuals which gained or lost weight under both ambient and high-temperature conditions. The goal of this research was to uncouple the influence of thermal stress from the effects elicited by poor growth performance and assess their relative contributions towards changes in blood parameter health indicators. Our specific objectives were to: (1) establish hematology and blood biochemistry reference data for *O.* *tshawytscha* presenting sub-optimal health, (2) utilise quantitative blood profiles to inform the mechanism and severity of the thermal stress response, and (3) gain functional insight into poor growth performance.

## 2. Results and Discussion

### 2.1. Survival and Growth

**Figure 1:**
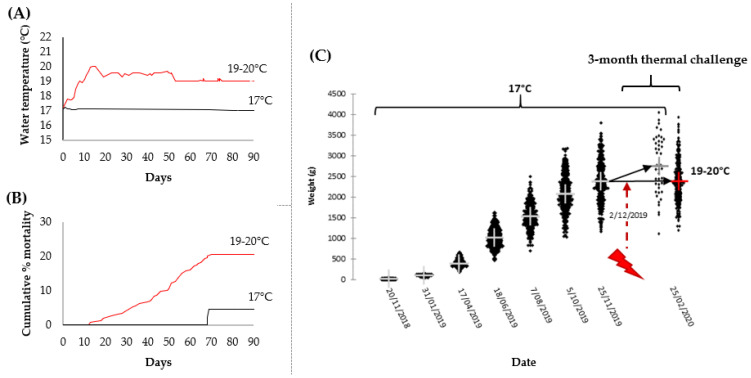
Effects of a chronic three-month thermal challenge (17 °C versus 19–20 °C) on *Oncorhynchus* *tshawytscha* performance. (**A**) Water temperature data for the thermal regimes (plotted as daily averages); (**B**) cumulative percent mortality during the challenge period; (**C**) total bodyweight of fish over the preceding one year of grow-out at 17 °C, and in the two temperature treatment groups on completion of the trial (individual fish are shown as dots with crosses representing their group means); the red lightning symbol indicates initiation of the thermal ramping to 19–20 °C in the temperature-challenged group.

A three-month thermal challenge at 19–20 °C resulted in 20% cumulative mortality and hampered the overall growth of surviving fish (Figure 1). These results align with performance metrics recorded by farmers during recent marine heatwave events [8], reinforcing that 19–20 °C approximates the chronic thermal maxima for this species (reared in seawater for a size range from 1 to 4 kg). Although, fish size, life stage, and stress history will influence this. Overall, mean body weight in the challenged group was similar pre- and post-thermal treatment. However, high variation in performance under both temperature regimes was highlighted by positive and negative specific growth rates (SGR). This trend afforded the opportunity to focus attention on divergent growth performance phenotypes decoupled from specific thermal stress-induced effects through selective sampling. Representative fish which gained or lost weight under each of the temperature treatments were nominated for targeted assessments of 13 cellular-based and 34 plasma biochemical parameters. See Supplementary Material Tables S1 and S2 for biometric information on selected candidates and statistical analysis outputs, respectively.

### 2.2. Thermal Stress Associations

**Figure 2:**
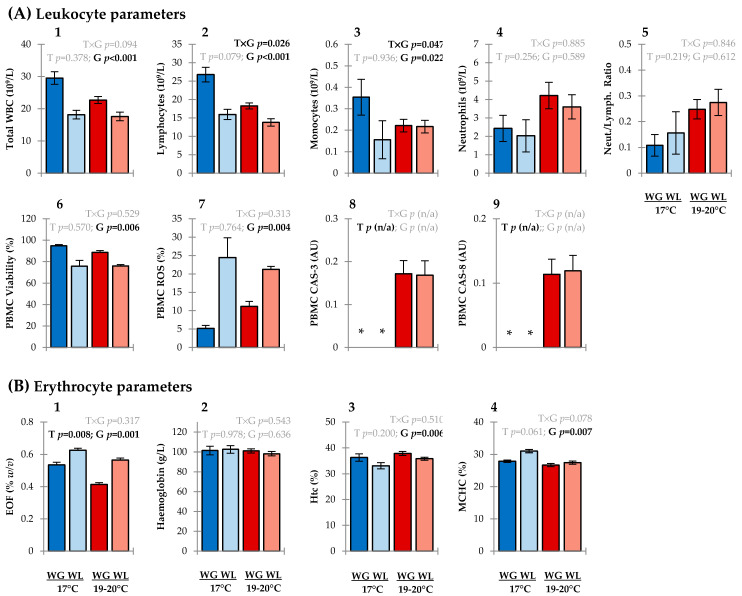
Cellular-based blood parameters associated with chronic thermal stress (17 °C vs. 19–20 °C) and growth performance (weight gain (WG) vs. weight loss (WL) phenotypes) in *Oncorhynchus tshawytscha*. (**A**) Leukocyte parameters; (**B**) Erythrocyte parameters. Data represent group means ± SE. Blue and red bars represent 17 °C and 19–20 °C treatments, respectively; darker and lighter shaded bars represent groups that gained and lost weight, respectively. Main effects of temperature (T), growth (G), and their interaction (T × G) are presented via their *p*-values in the upper section of each plot (generalized linear mixed models; α = 0.05). Asterisks signify non-detectable levels (statistical testing of these parameters are not applicable (n/a)).

Regarding leukocyte parameters, a main effect of temperature was not detected on total white blood cells (WBC) or differential leukocyte counts, neutrophil/lymphocyte ratios, viability of peripheral blood mononuclear cells (PBMCs (i.e., monocytes + lymphocytes)), or PBMC production of reactive oxygen species (ROS) (Figure 2A: 1–7). However, higher rearing temperatures triggered activation of caspase enzymes in PBMCs (Figure 2A: 8–9). Caspase (-3/-8) activities were non-detectable in PBMCs of fish reared under ambient temperature but were detected in their thermally stressed counterparts. Caspases are highly conserved proteases crucial to mediating programmed cell death, or apoptosis [19]. Caspase-8 is an initiator enzyme that regulates immune gene expression and the activation of downstream caspases, whereas Caspase-3 is an executioner enzyme responsible for the cleavage of intracellular proteins and fragmentation of DNA. The apoptotic cascade cumulates in the self-elimination of surplus or injured cells through chromatin condensation and nuclear fragmentation. Apoptosis is considered a ‘clean’ type of cell death whereby the cell membrane is left intact, and, unlike cell necrosis, there is no consequential inflammatory response [20]. In agreement with these results, there was no evidence to suggest that PBMC membrane integrity had been compromised (i.e., the non-discernible difference in PBMC viability through the dye exclusion principle). Upregulated transcription of various pro-apoptotic genes, including caspases, have previously been established as key thermal stress responses in *O. tshawytscha* [21]; our results complement these findings at the functional enzyme level.

Temperature also had an impact on erythrocyte osmotic fragility (EOF) (Figure 2B: 1). The lower EOF values in fish acclimated at the higher temperature for each of the growth performance groups indicates an increase in cellular membrane stability of erythrocytes. These results align with the inverse thermal-EOF relationship previously demonstrated in carp [22]. A mechanistic basis may correspond to differences in cell membrane composition. Erythrocyte membrane strength in fish is strongly influenced by the unsaturation index of the polar lipid component; higher incorporation of omega-3 fatty acids in phospholipids promote changes in the lamellar packing of the membrane and alters viscosity and fragility [23]. Water temperature was not associated with variations in erythrocyte haemoglobin concentration, haematocrit (Hct) values (i.e., packed cell volume), or mean corpuscular haemoglobin concentration (MCHC) (Figure 2B: 2–4).

**Figure 3:**
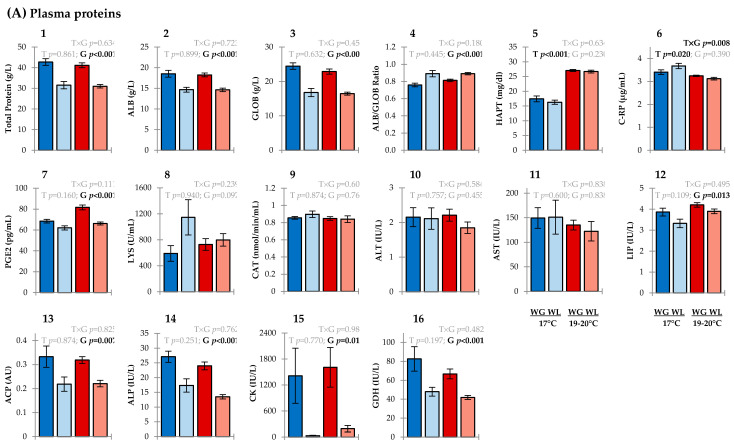

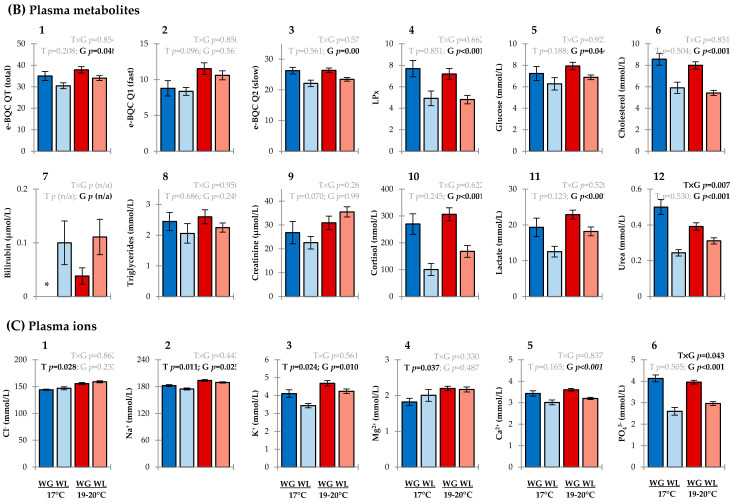
Plasma-based biochemical parameters associated with chronic thermal stress (17 °C vs. 19–20 °C) and growth performance (weight gain (WG) vs. weight loss (WL) phenotypes) in *Oncorhynchus tshawytscha*. (**A**) Protein parameters; (**B**) Metabolite parameters; (**C**) ion parameters. Data represent the group means ± SE. Blue and red bars represent 17 °C and 19–20 °C treatments, respectively; darker and lighter shaded bars represent groups that gained and lost weight, respectively. Main effects of temperature (T), growth (G), and their interaction (T × G) are presented via their *p*-values in the upper section of each plot (generalized linear mixed models; α = 0.05). Asterisks signify non-detectable levels (statistical testing of these parameters are not applicable (n/a)).

Although dominant proteinaceous plasma constituents (i.e., total protein, albumin (ALB), globulin (GLOB)) and ALB:GLOB ratios were not influenced by the main effect of temperature (Figure 3A: 1–3), increased levels of haptoglobin (HAPT) signalled a distinct immune response to the thermal stress treatment (Figure 3A: 5). Acute-phase proteins (APPs), such as HAPT, are potential biomarkers of trauma or infection in fish. Local inflammatory cells secrete cytokines (e.g., IL-1, IL-6, TNF-α) in response to tissue damage which stimulates hepatocytes to produce APPs and release them into circulation [24]. In salmonids, gradual increases in water temperature are reported to influence the transcription of APP-encoding genes [25,26]. However, when considering broader variations in the plasma protein panel, the limited effects on other key inflammatory-related markers such as total WBC counts (Figure 2A: 1) and levels of ALB (Figure 3A: 2), c-reactive protein (C-RP) (Figure 3A: 6), prostaglandin E2 (PGE2) (Figure 3A: 7), and lysozyme (LYS) (Figure 3A: 8) indicate a relatively mild immune response to the warmer conditions. Similarly, many of the immune-based plasma parameters in fish under both thermal regimes were within the normal reference range for healthy farmed Chinook salmon that we previously established [27].

No evidence of excessive redox imbalance due to thermal stress was uncovered via relative production of ROS in PBMCs (Figure 2A: 7), plasma activity of catalase (CAT) (a ROS-detoxifying enzyme) (Figure 3A: 9), plasma antioxidant capacities (eBQC values) (Figure 3B: 1–3), or through enhanced levels of lipid peroxides (LPx) (an oxidative damage biomarker) (Figure 3B: 4). The limited influence of temperature on activities of alanine aminotransferase (ALT), aspartate aminotransferase (AST) and lipase (LIP) enzymes suggests that the chronic thermal regime applied was unlikely associated with liver, bone, or pancreatic damage in surviving fish (Figure 3A: 10–12). Low thermal impact on numerous other plasma proteins (Figure 3A: 13–16) and metabolites (Figure 3B: 5–12) indicate robust homeostatic regulation of various physiological processes.

The levels of four plasma ions (Cl^−^, Na^+^, K^+^, Mg^2+^) were slightly elevated (Figure 3C: 1–4) in *O. tshawytscha* acclimated at 19–20 °C, indicating a shift in osmoregulation. Gill chloride cells utilize a Na^+^ gradient (established by a gill Na^+^/K^+^-ATPase in the basolateral membrane) to secrete Cl^−^ and uptake Na^+^ from saltwater to regulate plasma osmolality [28]. Diminished capacity for ion regulation in Atlantic salmon (*Salmo salar*) and sea bream (*Sparus aurata*) during thermal stress is associated with reduced Na^+^/K^+^-ATPase activity [29,30]. Evaluating our results beyond relative differences, chronic thermal stress-triggered plasma levels of Cl^−^ and Na^+^ to rise outside the normal reference range [27], indicating the severity of the effect. A further appraisal is needed to ascertain whether these ion levels were being maintained under homeostatic control and within an adaptive regulatory capacity.

### 2.3. Growth Performance Associations

Fish with highly divergent growth phenotypes presented markedly different haematological (Figure 2) and plasma biochemical profiles (Figure 3) irrespective of culture temperature. Twenty-eight out of the 47 blood parameters assessed were influenced by growth performance. Fish that lost weight during the experiment likely had reduced appetite and/or had stopped feeding. This is supported by the positive correlation (*r_s_* = 0.57; *p* < 0.001) of the feed intake at sampling versus weight gain/loss during the trial, differences in the hepatosomatic index (Supplementary Material Table S1), and the collective responses of plasma clinical biomarkers.

Classical signatures of long-term starvation in fish include reduced plasma levels of total protein, glucose, and cholesterol [31], all of which were significantly lower in the weight-loss group (Figure 3A: 1 and Figure 3B: 5–6). Complementing these findings were very low levels of creatine kinase (CK) (Figure 3A: 15) indicative of reduced ATP turnover and muscle weakening/deterioration, [12,32,33], lower activities of acid and alkaline phosphatase (ACP, ALP) (Figure 3A: 13–14) pointing towards malnutrition and impaired energy metabolism [34,35,36], and higher levels of bilirubin (Figure 3B: 7) symptomatic of liver dysfunction and/or starvation [37,38,39]. Interestingly, visceral fat scores (Supplementary Material Table S1) and circulating levels of triglycerides (Figure 3B: 8) were similar between growth performance groups, whereas the hepatosomatic index (Supplementary Material Table S1) was reduced in the fish that had lost weight which suggests differential utilization of lipid-based reserves during reduced calorie intake.

Cellular-based blood biomarker signals associated with starvation in fish also include symptoms of suppressed immunocompetence in the weight-loss group, as evidenced by lower total WBC counts (leukopenia) (Figure 2A: 1) and reduced viability of PBMCs (Figure 2A: 6). Such characteristics increase the risk of infections from pathogens [40]. Coupled with the diminished viability of these white blood cells was an increase in ROS production (Figure 2B: 7), revealing that PBMCs were also under enhanced oxidative pressure (potentially as an underpinning mechanism of necrosis). Lower total antioxidant capacity (TAC) (i.e., e-BQC QT values) in plasma compliments these data and provides further evidence of redox system imbalance in the weight-loss group (Figure 3B: 10). According to the relative e-BQC Q1/Q2 values (Figure 3B: 11–12), primary drivers for these growth performance-associated variations in TAC are endogenous metabolites with slow-reacting antioxidant behaviour (e.g., polyphenols). Lipid peroxidation products were diminished in the weight-loss group (Figure 3B: 4) which may be explained by fish having lower metabolic activities and/or through ketogenesis of protective metabolites (e.g., butyrate-derived metabolites, acetoacetate) under calorie restriction [41,42].

Cortisol and lactate are routinely used biomarkers for stress in fish and were detected at relatively high [8] concentrations in both growth performance groups (Figure 3B: 10–11). We attribute this to the crowding prior to handling and euthanisation via anaesthetic overdose. An initial aversion response to the active compound stimulates the hypothalamic–pituitary–interrenal (HPI) axis leading to cortisol accumulations; rising lactate levels follow as a secondary stress response [43,44,45,46]. Relative differences between growth performance groups were however still attributable. The lower cortisol levels in fish with negative growth may indicate exhaustion of the HPI axis as a response to one or more previously experienced persistent stressors [47,48]. The endocrine-based regulation of feeding and weight appears to be tightly coupled with associations between stress factors, corticotropin-releasing factor, and appetite [49,50].

Glutamate dehydrogenase (GDH) reversibly catalases the oxidative deamination of glutamate to produce α-ketoglutarate (as an energy substrate for the Krebbs cycle) and ammonia, playing a key role in nitrogen excretion in fish [51]. The lower GDH activity (Figure 3A: 16) (and mean urea levels (Figure 3B: 12)) in the weight-loss groups indicates limited protein catabolism. Our results contrast findings in slow- versus fast-growth phenotypes of Chinese perch (*Siniperca chuatsi*) being characterized by higher GDH activities [52]. Further interrogation of this mechanism in *O*. *tshawytscha* is warranted through experiments to interpret these data.

ALB and GLOB are two major components of plasma, which were deceased in the weight loss groups (Figure 3A: 2–3). ALB function in fish is not well understood whereas globulins are principal plasma proteins essential for maintaining immunocompetence. Levels of these proteins indirectly reflect the condition of specific humoral immunity and the ALB:GLOB ratio is widely used as an index of physiological state [53,54,55]. Considered non-specific biomarkers, lower protein levels can indicate a range of issues including liver and kidney dysfunctions, acute haemolytic anemia, infection, and inefficiencies in protein digestion and absorption [54,55,56,57]. ALB/GLOB ratios provide causative insight into lower total protein levels, and increased values observed in the weight loss groups (Figure 3A: 4) may indicate protein-energy malnutrition [58].

Altered levels of plasma ions (Na^+^, K^+^ Ca^2+^, PO_4_^3−^ (Figure 3C: 2–3, 5–6)) between the growth performance groups signal differential osmotic balance. Changes in the regulatory system of ionic interchange can alter blood pH and reduce erythrocyte volume, in turn affecting haematocrit (Hct) values [59]. In line with this, cellular parameters in fish with negative growth trajectories included lower percent Hct (Figure 1B: 3) possibly due to mild microcytic anemia [60], increased mean corpuscular haemoglobin concentration (MCHC) (Figure 2B: 4) suggesting a level of cell shrinkage and morphological degeneration [61], and higher erythrocyte fragility (EOF values) (Figure 2B: 1) indicating a weakening of the cell membrane [20].

### 2.4. Overview and Future Directions

In summary, we have established haematological and plasma biochemistry reference data for New Zealand farmed *O. tshawytscha* presenting suboptimal health in association with chronic thermal stress and divergent growth phenotypes. Uncoupling these influential factors on various biomarker responses allowed us to establish that water temperature in isolation triggered leukocyte apoptosis, altered erythrocyte membrane integrity, and provoked an inflammatory response; differential osmoregulation was a symptom of both factors. While these data suggest that fish that survived the 19–20 °C challenge were experiencing a low–moderate stress response, variable growth rates and relatively high incidences of mortality implicate more serious consequences on metabolism and wider physiological processes. Future characterization of metabolic differences, immune capacities, and genetic variation in individuals with very low thermotolerances (e.g., those which died during the thermal challenge) would help improve our understanding of mechanisms that support phenotypic differences.

Numerous blood biomarker signatures in fish with negative growth trajectories were clinically relevant to reduced feeding (substantiating their significance), and some provide insights to formulate and explore new hypotheses (e.g., roles of glutamate dehydrogenase, corticotropin-releasing factor, ketone bodies). Future studies involving elevated thermal stress in *O*. *tshawytscha* should incorporate temporal assessments of behaviour, metabolic rates, organ histology, compartmentalization/use of energetic reserves, and broad evaluations of endocrine target complements (transcript-to-metabolite). With specific relevance for aquaculture, it will also be important to establish whether there is an interdependency between thermal stress and resilience with growth performance and to determine if other pre-existing performance-based metrics (e.g., health indices, feed efficiency, energy reserves) are influential to an individual’s fitness in coping with elevated temperatures. Discerning such associations provide significant scope to improve farm management protocols and develop pre-emptive strategies before anticipated spikes in sea surface temperatures (e.g., identifying and moving/protecting susceptible stock). Our results reveal broad variability in thermal resilience among individuals, supporting possibilities for trait enhancement through genetic selection or other means. Heritable and non-heritable mechanistic bases underpinning variability in thermotolerance are recommended areas for the aquaculture industry to capitalize on selective breeding potential and to assist advances in husbandry-based practices. The building of robust and resilient salmon will help wild and domesticated fish stocks to withstand global change, and extend opportunities for aquaculture development.

## 3. Material and Methods

### 3.1. Experimental Design and Sampling

Smolts from the breeding programmes of two commercial companies (Sanford Ltd. (Auckland, New Zealand) and New Zealand King Salmon (Nelson, New Zealand)) were tagged with passive integrated transponders, transferred to the Cawthron Finfish Research Centre (FRC) (Nelson, New Zealand), and maintained at 17 °C on a saltwater recirculatory aquaculture system (RAS). Fish were fed to satiation daily with a standard commercial diet (Skretting; Hobart, Tasmania, AUS) (see Supplementary Material Table S3 for the proximate composition of feed pellets). After one year of rearing (from Nov 2018 to Nov 2019), 329 fish (mean weight ± SD (g) = 2103 ± 443; mean fork length ± SD (mm) = 451 ± 26) were randomly distributed among 12 × 500 L tanks. Ten tanks were assigned to a high-temperature treatment (for evaluation of thermotolerance trait heritability); during Dec 2019, the water temperature was gradually increased from 17 °C to 20 °C at 0.13 °C per day for 23 days. The other two tanks were utilised as in situ controls (17 °C). Finetuning of the high thermal condition was conducted during the first few weeks of the experiment based on reduced feeding behaviour and incidences of mortality, with a final thermal challenge temperature being reduced to 19 °C. Levels of dissolved oxygen within elevated-temperature treatment tanks were also manipulated (lowered) to mimic conditions of the natural marine environment during warmer summer periods (Supplementary Material Table S4). Fish were fed to satiation once daily, and mortalities recovered. The entire thermal challenge experiment ran for 86 days. At the conclusion of the experiment towards the end of Feb 2020, feed intake assessments were made on all surviving fish (*n* = 267) using the Ballotini method via X-ray [62,63,64]. Fish were individually weighed and selectively sampled to provide representative numbers of individuals which gained weight and lost weight from every tank across the two thermal treatments. Depending on the numbers of fish available, 5–16 fish from each of the 12 tanks were obtained for blood extraction; an unbalanced sampling regime was consequently implemented with biological replication for each of the fixed effects (temperature, growth) ranging from 10–60 fish (see Supplementary Material Table S5). After being x-rayed, weighed, and assigned a growth performance group, fish were transferred to a holding tank with a divider down the middle to keep the groups separate during a recovery phase; the densities of fish in the holding tank were never higher than their rearing density. Fish were individually captured from tanks via scoop net, euthanised by anaesthetic overdose (AQUI-S^®^; 80 ppm; 7 min), and 3–4 mL of peripheral blood was withdrawn from the caudal vein. Sub-aliquots of blood were allocated for analyses of various cellular haematology and plasma biochemistry parameters.

### 3.2. Biometrics

All fish assigned for blood analyses were evaluated for biometric parameters comprising: whole body weight (BW (g)), fork length (FL (mm)), girth (mm), Fulton’s condition factor (CF) (i.e., (BW × 10^5^)/FL^3^), specific growth rate (SGR (%BW gain per day during the three-month challenge period)), swim bladder fluid (SBF (mL)), stomach width (mm), gastrointestinal tract weight (g), visceral fat score (VFS (1–4 scale)), belly-flap thickness (in three locations: cranial to the pectoral fin, caudal to the pelvic fin, at the vent), cardiosomatic index (heart weight/BW), hepatosomatic index (liver weight/BW), and gonadosomatic index (gonad weight/BW).

### 3.3. Cellular-Based Parameters

Differential cell counts were evaluated using two whole blood smears per fish. Slides were air-dried and transported to an accredited laboratory (Gribbles Veterinary; Christchurch, New Zealand) for staining (Leishman) and processing within 72 h of collection. Leukocyte counts (i.e., white blood cells) were estimated based on the average counts of 10 fields. Data are presented as a range and absolute values of the differential are estimated based on the mean of this range. Differential leukocyte counts were manually determined based on a count of 100 cells. Absolute values for lymphocytes, neutrophils, and monocytes were determined from the fraction of the 100-cell differential multiplied by the mean of the leukocyte count range.

Mean corpuscular haemoglobin concentrations (MCHC) were calculated from Hb contents and haematocrit (Hct) values [60,65]. For haemoglobin content, 50–100 µL of whole blood were transferred to a 1.3 mL BD-Microtainer^®^ tube containing lithium heparin (Becton Dickinson; Franklin Lakes, NJ, USA), stored at 4 °C, and analysed by Gribbles Veterinary laboratory within 48 h of collection using a Hb201 + system (HemoCue^®^; Angelholm, Sweden). The Hct values, measured as packed cell volume, were determined on whole blood immediately after withdrawal using heparinised micro haematocrit capillary tubes (Kimble Chase). Tubes were centrifuged (9000 rcf; 7 min) in a microhaematocrit centrifuge.

Erythrocyte osmotic fragility (EOF) was measured following established protocols [66]. A series of saline solutions were prepared to provide NaCl concentrations of 0.00, 0.10, 0.20, 0.40, 0.60, 0.80, and 0.85% *w/v* in distilled water. 10 μL of whole blood which had been collected in lithium heparin BD-Microtainers were mixed thoroughly with 2.0 mL of the ascending series of NaCl solutions. Suspensions were incubated at room temperature for 30 min then centrifuged (900 rcf; 10 min). The optical density of the supernatant was determined spectrophotometrically at 540 nm with distilled water as a blank. The percentage haemolysis is expressed relative to the solution having the highest optical density reading (i.e., maximum haemolysis). Mean corpuscular fragility (MCF), or the EC_50_ value (effective concentration causing 50% haemolysis), was determined using the equation derived from a four-parameter dose-response curve: x=c ((a−d)/(y−d)−1)^(1/b); where *a* = minimum value of the fitted sigmoid curve; *b* = Hill’s slope of the curve; *c* = point of inflection; *d* = maximum value of the fitted sigmoid curve; *x* = MCF (EC_50_); and *y* = 50 (% haemolysis).

Peripheral blood mononuclear cell (PBMC) isolation and purification was performed using our established protocol [67]. Briefly, 284 µL of whole blood (collected in lithium heparin BD-Microtainers^®^) was diluted 1:1 with sterile filtered (Whatman 40 µm (Cytivia; Vancouver, BC, Canada)) PBS (SF-PBS) (pH 7.4) and centrifuged (971 rcf; 20 min) over a layer of 682 µL Histopaque sterile filtered density gradient medium (10771-6 (Sigma-Aldrich; Auckland, New Zealand)) in 1.5 mL Eppendorf tubes. Cells at the interface were aspirated with a pipette and washed twice in 500 µL of SF-PBS (being centrifuged between [674 rcf; 7 min]). The resulting PBMC pellets were re-suspended to final cell concentrations of 10^5^–10^6^ cells mL^−1^ in SF-PBS supplemented with 2% fetal bovine serum and immediately processed for PBMC viability and ROS production.

PBMC viability assessments were performed via flow cytometry (Muse^®^ Flow Cell Analyzer (Merk; Darmstadt, Germany)) and a commercial assay kit (Muse^®^ Cell Count and Viability Kit (Merck)) using validated protocols for *O. tshawytscha* leukocytes [67]. To first establish correct Muse^®^ gating parameters, a PBMC sample (10^5^–10^6^ cells mL^−^^1^) from a single fish was used to prepare two matched suspensions of live versus dead cells. PBMC death was quickly induced by adding 10 μL of diluted (1:100) Trigene detergent to one of the suspensions. 20 μL of each suspension was subsequently mixed with 380 μL of assay reagent, vortexed, and incubated at 18 °C for 5 min before being analysed via flow cytometry. Cell size gating and viability thresholds were checked based on these data and set for subsequent sample analyses. Purified PBMC samples of experimental fish were similarly analysed, without the addition of detergent.

Intracellular ROS production (i.e., specifically superoxide anions) in PBMCs was measured via flow cytometry (Muse^®^ Cell Analyzer) using a commercial assay kit (Muse^®^ Oxidative Stress Kit (Merk)). Minor modifications to the manufacturers’ protocol were implemented to account for the normal physiological temperature range of the fish model: 20 μL of PBMC suspension were incubated for 30 min at 18 °C with 180 μL of Muse^®^ Oxidative Stress working solution prior to analysis [68]. To first establish correct Muse^®^ gating parameters, a PBMC sample (10^5^–10^6^ cells mL^−^^1^) from a single fish was used to prepare two matched calibration suspensions of cells with differing ROS profiles. ROS production was quickly induced in one of these samples by adding 10 μL of 2 mM menadione:EtOH solution to 190 μL of PBMC suspension and incubating at 18 °C for 30 min. Calibration suspensions were stained as above with assay kit reagents and their ROS profiles were used to set the gating and threshold parameters for subsequent sample analyses.

Caspase-3 and -8 activities in PBMCs were measured colorimetrically using commercial assay kits (ab39401 and ab39700 (Abcam; Cambridge, MA, USA]) with minor modifications for frozen samples. Briefly, fish PBMCs (10^5^–10^6^ cells mL^−^^1^) were lyophilised overnight at −75 °C. Samples were re-suspended in 300 μL of chilled cell lysis buffer, incubated on ice for 10 min, then centrifuged (10,000 rcf; 1 min; 2 °C). Total protein contents of supernatants were first quantified using a commercial assay kit (ab102536 (Abcam)) following the manufacturers’ instructions, and each sample was adjusted to 50–200 µg protein per 50 μL cell lysis buffer. Duplicate samples and controls were prepared on a 96-well microplate and protein content was measured at 562 nm against a bovine serum albumin standard curve. For caspase enzyme activity, reactions (96-well microplate) comprised 50 μL of sample or blank (2x reaction buffer) in duplicate, 50 μL of the caspase reaction mix, and 5 μL of 4 mM DEVD-pNA substrate (DEVD-pNA or IETD-pNA (200 μM final concentration)). Plates were incubated at 37 °C for 1 h, and absorbances were measured at 405 nm using a UV-vis microplate reader (Multiskan^TM^ Go (Thermo Scientific; Vantaa, Finland)). Data were normalized to total protein.

### 3.4. Plasma Biochemistry Parameters

Fresh peripheral blood samples were centrifuged (16,250 rcf; 8 min) to obtain plasma, transferred to 2 mL sterile cryovials (Interlab; Auckland, New Zealand) and immediately snap-frozen in liquid nitrogen. Subsamples were sent on dry ice to Gribbles Veterinary laboratory for targeted and quantitative analyses of selected biochemical and haematology parameters. Biochemical analytes comprised six ions (K^+^, Na^+^, Mg^2+^, Ca^2+^, Cl^−^, PO_4_^3−^), seven metabolites (urea, creatinine, lactate, glucose, cortisol, bilirubin, cholesterol), total triglycerides (TAGs), total protein (TP), albumin (ALB), globulin (GLOB (calculated as TP minus ALB)), haptoglobin (HAPT), prostaglandin E2 (PGE2), C reactive protein (C-RP), and six enzymes (alkaline phosphatase (ALP), alanine aminotransferase (ALT), glutamate dehydrogenase (GDH), creatine kinase (CK), and lipase (LIP). Plasma samples were analysed for electrolytes and most clinical chemistries using an automated chemistry analyser (Cobas c 501 (Roche Diagnostics, Mannheim, Germany)). Plasma cortisol levels were determined using an automated endocrinology analyser (Cobas e 411 (Roche Diagnostics, Mannheim, Germany)). Each of the assays used a standard kit (Roche Diagnostics, Mannheim, Germany) developed for the autoanalyser. The inflammatory markers HAPT, C-RP and PGE2 were analysed in plasma samples using ELISA kits (My BioSource; CA, USA) and read on a SpectraMax^®^ ABS microplate reader (Molecular Devices; CA, USA).

Lysozyme (LYS) activity was assessed spectrophotometrically [69]. Briefly, 200 μL *Micrococus lisodeikticus* (M3770 (Sigma-Aldrich; Auckland, New Zealand)) suspension (0.2 mg/mL in 0.05 M PBS) were added to wells of a 96-well microplate and mixed with 50 µL of freshly thawed plasma. Absorbance (520 nm) was recorded after 1, 3, 6, and 9 min with shaking in between (Multiskan^TM^ Go (Thermo Scientific)). One unit of enzyme activity was defined as the volume of sample required to cause a 0.001/min decrease in absorbance from the slope of the linear portion of the curve.

Acid phosphatase (ACP) activity was assessed spectrophotometrically [70]. Fish plasma was thawed and used without further processing. To 80 µL of sample in a well, 20 µL of 0.2 M, pH 5.0 acetate buffer (S7899 (Sigma-Aldrich; Auckland, New Zealand)) and 2 µL of 24 mM, pH 5.0 para-nitrophenyl-phosphate (N7653 (Sigma-Aldrich; Auckland, New Zealand)) were added. The suspension was incubated for 30 min at 12 °C. After 30 min, 200 µL of 0.2 M borate buffer (pH 9.8) were added to stop the reaction and absorbance was recorded at 405 nm (Multiskan^TM^ Go (Thermo Scientific)). Results were recorded in absorbance units.

Catalase activity was determined using a colorimetric assay kit (ab83646 (Abcam)) according to the manufacturer’s instructions. In this assay, catalase decomposes H_2_O_2_ to water and oxygen, and then unconverted H_2_O_2_ reacts with OxiRed probe to produce a product that can be measured at 570 nm. Briefly, plasma samples in a 96-well plate were treated with H_2_O_2_, incubated for 30 min at 21 °C, then followed by addition of stop solution and development solution. The absorbance was measured on a microplate reader (Multiskan^TM^ Go (Thermo Scientific)) and results were expressed as nmol H_2_O_2_ converted per min per mL of plasma.

Lipid peroxidation was determined using a colorimetric assay kit (KB03002 (Bioquochem; Oviedo, Spain)) according to the manufacturer’s instructions. This assay measures two major by-products of lipid peroxidation: malondialdehyde (MDA) and 4-hydroxynonenal (4-HNE). The reaction between MDA and HNE and the assay reagent A results in a chromophore (diindolylalkane) with maximum absorbance measured at 586 nm. Salmon plasma samples were thawed on ice and 100 µL mixed thoroughly with 325 µL of Reagent A solution and 75 µL of Reagent B in a 1.7 mL microcentrifuge tube. Tubes were incubated in a water bath at 40 °C for 40 min, then centrifuged (5000 rcf; 5 min; 21 °C). 200 µL of supernatant from each sample were aliquoted in duplicate into wells of a 96-well plate together with calibration standards (0–60 µM). Absorbance was measured at 586 nm (EnSpire^®^ microplate reader (PerkinElmer^®^)). MDA and HNE concentrations were calculated using the sample absorbance (blank corrected) and the slope from the linear regression of the standard curves (R^2^ = 0.9902–0.9996).

Total Antioxidant Capacity (TAC) was assayed using an e-BQC portable TCA device (Bioquochem; Oviedo, Spain) with disposable strips which determines antioxidant capacity using electrochemistry; results obtained are in micro-Coulomb (µC). A 50 µL sample of chilled plasma was placed onto a disposable strip, obtaining results for fast (Q1) and slow (Q2) antioxidant responses as well as total antioxidant response (Qt: sum of Q1 and Q2).

### 3.5. Statistical Analyses

General linear mixed models (GLMMs) were used to assess the effects of thermal stress, growth performance, and their interaction on all cellular haematology and plasma biochemistry parameters, with the tank being included as a random factor where possible. Individual MCF values were used as a measure of erythrocyte osmotic fragility (EOF). Analysis of PBMC viability and ROS production was similarly performed but with ‘time-to-analysis-after-blood-withdrawal’ being included as a potential covariate due to variability in sample processing times (mean ± SD = 105.7 ± 28.2 min). Data were analysed using XLSTAT v2021.2.1 statistical software (Addinsoft; New York, NY, USA).

## Data Availability

The data that support the findings of this study are available on reasonable request from the corresponding authors. The data are not publicly available due to privacy.

## Supplementary Materials

The following are available online at https://www.mdpi.com/article/10.3390/metabo11080547/s1, Table S1: Biometric information of selected study candidates, Table S2: Effects of temperature and growth performance on Chinook salmon (*Oncorhynchus tshawytscha*) cellular- and plasma-based blood parameters, Table S3: Proximate composition of feed pellets used prior to and during the experimental period, Table S4: Average daily levels of dissolved oxygen during the experimental period, Table S5: Sample numbers.
